# Professionalism lapses in health professions training: Navigating the ‘Yellow Card’ moments for transformative learning

**DOI:** 10.1111/medu.15540

**Published:** 2024-10-02

**Authors:** Matt Sibbald, Urmi Sheth, Nicole Last, Amy Keuhl, Isla McPherson, Sarah Wojkowski, Dorothy Bakker, Paula Rowland

**Affiliations:** ^1^ Michael G. Degroote School of Medicine McMaster University Hamilton Ontario Canada; ^2^ Educational Services, Faculty of Health Sciences McMaster University Hamilton Ontario Canada; ^3^ Department of Family Medicine, Faculty of Health Sciences McMaster University Hamilton Ontario Canada; ^4^ School of Rehabilitation Science, Faculty of Health Sciences McMaster University Hamilton Ontario Canada; ^5^ Program for Interprofessional Practice Education and Research, Faculty of Health Sciences McMaster University Hamilton Ontario Canada; ^6^ Department of Occupational Science and Occupational Therapy, Temerty Faculty of Medicine University of Toronto Toronto Ontario Canada; ^7^ Centre for Advancing Collaborative Healthcare & Education, Temerty Faculty of Medicine University of Toronto Toronto Ontario Canada; ^8^ The Institute for Education Research University Health Network Toronto Ontario Canada

## Abstract

**Introduction:**

Health professions training programmes face increasing reports of professionalism lapses, which can delay, or end, trainee progression. How programmes respond to professionalism lapses to facilitate professional identity development has not been clarified. The objective of this study is to identify factors that facilitate and impair transformations around professionalism lapses in health professions training programmes.

**Methods:**

We conducted a qualitative study interviewing 5 faculty and 20 trainees with firsthand or secondhand experience with professionalism lapses from a range of health professions training programmes at McMaster University. Using reflexive thematic analysis, we coded verbatim transcripts informed by the lenses of social and transformative learning theories. We constructed themes through iterative and comparative analysis, seeking meaningful variation across professions and triangulating faculty and trainee perspectives.

**Results:**

Four themes were constructed. First, lapses are in the eye of the beholder with personal definitions intersecting with institutional and situation norms. Difficulties exist in recognising and convincing trainees to respond to lapses that are perceived to be minor or subject to interpretation. Second, responses to professionalism lapses occurred within power hierarchies, which impacted how trainees reacted to the remediation process, risked superficial trainee responses to concerns and led to concerns around inequitable treatment in how standards were applied. Third, fostering transformation involves building trainee confidence, agency, trust and engagement. Focused support and advocacy for trainees can empower and promote agency in tackling disorienting lapses. Fourth, perspective shifts involve deep engagement over time, including but not limited to self‐reflection, structured discussion and seeking support.

**Discussion:**

Identifying and addressing professionalism lapses is complex and requires nuanced and contextual exploration of personal, institutional and situational dynamics at play. By fostering environments that promote genuine reflection and dialogue and focus on building trainee confidence, agency, trust and engagement, health professions training programmes can better support trainees in navigating these complex situations and contribute to the broader goal of socialising to a professional culture and practice.

## Abstract 2

Addressing professionalism lapses with healthcare trainees is shown to contextualize the varied perceptions around lapses that occur due to personal, institutional, and situational factors.

## 1 INTRODUCTION

Professionalism requires a complex, contextual integration of knowledge, skills and attitudes that culminate in professional identity.^1^ Developing professional identity is a longitudinal, transformative process^2^ and an outcome of all health professions training programmes. Consistently meeting this outcome is not easy; trainees are tasked with layering knowledge and understanding of the practice of a profession onto their personal identities and social‐cultural contexts. These sociocultural contexts, along with changing norms around professionalism, have created increasing rifts between the worlds that many trainees come from and the communities of healthcare providers they seek entry into. This growing gap challenges training programmes to provide an increasing amount of support for professional identity formation,^3^ and continual pressure to evolve and re‐contextualise the definition of professionalism.^4^, ^5^

Much is at stake. As the rift widens, programmes face increasing reports of professionalism lapses in trainees—akin to ‘Yellow Card’ warnings in sports to change behaviour or receive progressive discipline. In turn, these professionalism lapses increasingly hold up, if not end, trainee progression in programmes. Certification bodies are increasingly raising concerns, as professionalism lapses during training predict disciplinary action in practice.^6^

What is needed is an understanding of how healthcare training programmes can best support trainee transformation when professionalism lapses are identified.^7^ We recognise professionalism is learned through mistakes^8^, ^9^ and role modelling.^10^, ^11^ We realise lapses often signal personal stressors,^12^ burnout, illness and complex social‐cultural contexts.^13^, ^14^ What has yet to be clarified is how programmes should respond to professionalism lapses to facilitate trainee transformation.

Two theoretical traditions shed light on this process. The first is a cluster of theories binding sociology to learning: situated learning,^15^ experiential learning,^16^ communities of practice^17^ and landscapes of practice.^18^ These theories position learning as a social endeavour requiring genuine peripheral participation in a health professions community to build identity through shared experiences, sensemaking and recognition of the self as a health professional. Social learning theories are particularly relevant to professionalism lapses as they emphasise the importance of context, interaction and community where lapses are identified and codified. Professionalism norms and values are shared and negotiated within healthcare communities of practice, with professionalism learned and reinforced through genuine trainee participation in these communities.^17^ Similarly, Lave and Wenger^15^ describe situated learning as active engagement in authentic contexts, providing a frame to understand how trainees internalise professional behaviours and attitudes. This perspective is supported by Durning and Artino^19^ who argue professional identity and behaviour are shaped by the social environments in which learners are immersed. Essentially, this focus on the social aspects of learning draws attention to the ways in which people make meaning from their social contexts and draw from these meanings in order to construct their identities.^20^ By understanding trainee professionalism lapses through the lens of social learning theories, the contextual and relational factors that influence these behaviours can be defined and clarified with the goal of providing better support to trainees.

The second theoretical tradition is transformative learning itself, a process of deliberate self‐examination focused on changing one's perspective to enable fundamental change of self.^21^, ^22^ This process is primed by ‘disorienting dilemmas’—threats to identity and self—which, when adequately supported, can lead to transformation through critical reflection, discourse and action.^23^ Prior work has identified factors that facilitate transformative learning in undergraduate clerkships^24^ and postgraduate education.^25^ In undergraduate clerkships, supportive learning environments, opportunities for reflective practice and strong collaborative relationships facilitated transformative learning. For example, Greenhill^24^ found that longitudinal integrated clerkships provide continuity in the learning environment thereby enhancing trainees' ability to engage deeply, reflect on their practice and develop transformed perspectives around professional identity. Similarly in postgraduate education, mentorship, feedback and creating safe spaces for critical reflection all facilitated professional transformation. Vipler^25^ emphasised the importance of mentorship in guidance and support, enabling trainees to navigate disorienting dilemmas effectively. Furthermore, structured feedback and opportunities for discourse with peers and mentors contributed to the transformative learning process, allowing trainees to critically examine their experiences and assumptions. In applying a transformative lens around professionalism lapses, whether a lapse leads to meaningful examination of assumptions or beliefs requires trainees to have sufficient support and structure to unpack a disorienting dilemma through critical reflection and discourse.

## 2 OBJECTIVE

The objective of this study was to identify factors that facilitate and impair transformations around professionalism lapses in health professions training programmes. Using the lenses of social and transformative learning theories, we unpack structures and processes to inform programmes on how best to support professional identity formation among trainees.

## 3 METHODS

We conducted a theory‐informed, exploratory study using semi‐structured interviews of key informants around how professionalism lapses are addressed in health professions training programmes at McMaster University.

### 3.1 Sampling and recruitment

We invited faculty involved in remediating professionalism and students with firsthand or secondhand experience with professionalism processes during their health professions training programmes within McMaster's Faculty of Health Sciences. We deliberately sampled across multiple health professional programmes including undergraduate medicine, postgraduate medicine, physician assistant, nursing, midwifery and rehabilitation sciences (i.e. physiotherapy, occupational therapy, speech language pathology) to identify common themes across professions, and unique dynamics specific to particular programmes. We deliberately sampled firsthand and secondhand accounts to provide information on how professionalism lapses were experienced and perceived by peers. Faculty were recruited through contacts in educational programmes on the study team (MS, IM, DB, SW). Trainees were made aware of the study by an email sent out from programmes. Those with firsthand or secondhand experience with professionalism processes were invited to contact a research coordinator who had no role within any of the programmes.

### 3.2 Interview process

Interviews were conducted virtually and recorded. Interviews were conducted by one health professional and one non‐health professional to facilitate a safe discussion space and ensure a sense of familiarity with the content domain but maintain sufficient distance to ensure trustworthiness of the analysis. Neither interviewer had a role within any healthcare training programme, thereby maintaining an objective perspective during the co‐construction of data.^26^ The interview guides (Appendix S1) focused on structures and processes around the management of professionalism lapses moving from open ended questions around how professionalism lapses are defined, uncovered, managed and remediated to probes around components relevant to social and transformative theoretical lenses (e.g. to what extent does critical reflection occur in the process). The interview process was interleaved with cycles of analysis to allow an iterative approach to sampling and a data‐informed evolution of the interview guide.

### 3.3 Analysis

Interviews were transcribed verbatim, and all material anonymized prior to analysis. We chose an approach informed by reflexive thematic analysis, as a theoretically flexible method suited to identifying patterns and themes across diverse professional programmes within a social constructivist paradigm. This approach allowed us to explore the data deeply and remain open to inductive analysis of themes from the data while considering deductively the social and transformative learning theoretical frameworks.^27^ Sensitising concepts from transformational learning included perspective shift or transformation itself, disorienting dilemmas, critical reflection, collaboration and implementation. Sensitising concepts from social learning theory included norms and values, role modelling, situational factors, cultural influences, peer interaction, mentorship, supervision, experiential learning and reflective practice. Analysis occurred in a staged approach moving from qualitative description^28^, ^29^ to theory‐informed focused coding.^30^ The analytic team met monthly in 1 h sessions for 1.5 years to iteratively review transcripts. The analytic team was provided 2–4 transcripts in advance of each meeting to facilitate familiarisation, memoing on shared documents and discussion. In keeping with reflexive thematic analysis, this created opportunity for analysis of both surface meaning (semantic analysis) as well uncovering covert and implicit meaning (latent analysis). Further, analytic meetings included reflection on participant sampling, interviewing techniques, tensions and discrepancies, as well as study team assumptions, values and beliefs. Initial readings relied predominantly on open coding to identify key themes, sensitised by the theoretical perspectives of social and transformative learning theories, in a manner consistent with theory‐informed focused coding^30^; this was followed by an iterative, axial approach to coding to provide more in‐depth linkages between material, searching for coherence and well‐supported conclusions. We continued to conduct interviews until the study team felt satisfied that there was robustness to the exploration of the issues, and meaningful themes for the health professions education community could be constructed. Guided by our study aims, a priori, we anticipated requiring 10–15 interviews to reach sufficient information power for our study aims.^31^

### 3.4 Analytic team

The analytic team comprised members from diverse professional backgrounds (occupational therapy, physical therapy, medicine, standardised patient trainer, teacher) with diverse roles including a learner (US), educators (IM, DB, MS, SW), researchers (MS, SW, PR, NL), leaders of educational programmes (MS, DB, SW), leader of a professionalism process (IM), a professionalism coach (DB) and the leader of a professionalism standards committee (DB). This diversity of professional backgrounds and roles enabled us to distance ourselves from the cultures and traditions of individual professions and programme processes. We also ensured there were perspectives outside of the educational programmes (NL, AK) and institution (PR) being studied to allow perspectives on internal hierarchies and power dynamics. We took a social constructivist stance, which acknowledges that knowledge and understanding are constructed through social interactions and shared experience. By adopting this stance, we aimed to understand how participants' experiences and interpretations of professionalism lapses were shaped by their interactions and the social environment of their training programmes.

### 3.5 Rigour

Rigour was built into data collection and analytic phases. Data collection rigour included deliberate seeking of meaningful variation and deviant cases for sampling,^32^ checking transcripts for accuracy and careful records of context for interviews. Analytic rigour included triangulating data from faculty and students, examining multiple theoretical perspectives, reflexive journaling by the analytic team, revisiting phases in the analytic process when required and constructing carefully of the analytic team to balance perspectives.^33^

## 4 RESULTS

Four themes were constructed from 25 interviews, 5 with faculty and 20 with trainees. Aggregated participant demographic information is shown in Table 1.

**TABLE 1:** Overview of participant demographic information, separated by trainee and faculty participants.

Demographic data	Number of participants
Trainees	
Programme of study	
Midwifery	2
Nursing	1
Postgraduate medicine	5
Physician assistant	2
Rehabilitation science	2
Undergraduate medicine	8
Professionalism process experience	
Firsthand	8
Secondhand	7
Both	5
Gender identity	
Female	11
Male	9
Race/cultural identity	
African	1
East Asian	4
Latin American	1
South Asian	6
Southeast Asian	1
White and/or European	7
Faculty	
Program	
Medicine	3
Rehabilitation	2
Gender	
Female	2
Male	2
Prefer not to answer	1
Involvement with trainee professionalism lapses (number of processes)	
1–2	0
3–9	3
10 or more	2

### 4.1 Theme 1: lapses are in the eye of the beholder

Personal definitions of ‘professionalism lapse’ widely varied among students and faculty, with a sense they were often influenced by subjective interpretations and expectations. These ranged from ‘blatant disregard for professional standards’ (Trainee 5) to subtler ‘grey areas and misunderstandings’ (Trainee 2), with a recognition that professionalism lapses could also be simply lapses in judgement or unintentional actions. The challenge lies in the varying interpretations of what constitutes a lapse. For instance, inappropriate comments or breaches of confidentiality can be clear lapses for one person but seen as minor or accidental by another.

The intersections between personal definitions and institutional norms and expectations are further nuanced by situational and contextual factors: ‘every institution or program or community has a set of professionalism guidelines’ (Trainee 13). As Faculty 1 noted, ‘Professionalism itself is contextual and dynamic and always changing’, while Trainee 17 observed, ‘… the perception of professionalism also comes from your personal background, your cultural background, and what you are used to before entering that space’. The variation in definitions and expectations, coupled with the importance of situational and contextual factors, makes shared understanding of lapses complex: ‘Some of the situations I have been involved in that I found the most challenging to manage have been with international medical graduates who have studied, trained, and some even worked in a different medical culture with different expectations’ (Faculty 1). This emphasises the difficulty in aligning diverse perspectives within a single set of standards.

The context bound nature of professionalism lapses contributed to challenges in recognising or acknowledging lapses, especially when implicit, inconsistent or part of a hidden curriculum around expected social and/or communication norms: ‘Sometimes those standards are explicitly communicated to you. But many times it may be informally applied based [on] social or cultural norms. And those may not always be articulated. Or they may be context or person specific’ (Trainee 15).

Sometimes you have inconsistent standards. So sometimes they will have attendings where they are totally fine with you calling them by their first name and joking around. And then there are other attendings who would really react negatively to that. So … you can get into a certain mode of … how you communicate with people, that doesn't always work when you are going from site to site with people who have kind of standards for how you should act. (Trainee 5)

### 4.2 Theme 2: power hierarchies shaded responses to professionalism lapses

Responding to lapses was a complex task, embedded within the challenge of a power imbalance between trainees and faculty that was difficult to separate from a punitive or disciplinary lens: ‘But it seems like it ends up being an “us versus you” situation. And they are trying to kind of either drop the hammer or trying to discipline you in a way’ (Trainee 1).

Questioning a professionalism lapse could also be viewed as evidence of unprofessionalism, a form of double jeopardy: ‘We don't really have any kind of ability to advocate for ourselves in the professionalism process because if you question the professionalism referral, that is in and of itself considered unprofessional’ (Trainee 5). Power dynamics can create a defensive stance among students:

It was reiterated to me over and over again that this process is for your own health and your own growth. And we are all trying to support you. And I am like, no it kind of sounds like you are accusing me of something. And I sort of have to defend myself. Sort of like a court hearing, I am the accused, right? And you guys are accusing me. And I have the legal counsel, a.k.a. my student advisor. (Trainee 2)

Faculty members also reflected on challenges of identifying and communicating lapses, given the inherent power differential between faculty and students:

But I think in some cases yes, they can be fear‐based …. And there were certain expectations of me as a student …. Or that there were certain ways that I should and shouldn't speak to [faculty] or converse with them. Just because of the nature of their position perhaps. (Faculty 3)

Faculty also perceived a risk that students will interpret discussion around professionalism lapses as personal attacks: ‘It made the student feel like there was an attack on their identity, and hence the response was to protect their identity and their values’ (Faculty 2).

Power struggles also arose from perceived differences in applying standards. For example, ‘The student came back with, “well these are the things that the clinical instructor is not doing well that are not maintaining professional boundaries”’ (Faculty 2), or, ‘Is this really necessary for this office? I personally have seen physicians look up their own charts … and nothing happens to them because they are staff’ (Trainee 3). Trainees also felt trapped by a hidden curriculum of workplace practices that deviated from professional expectations:

There is a lot of unique pressure in first year to do the same thing that your seniors do …. I always cycle back to the putting names in chats example. Like lots of seniors will do that, but I know that is not allowed. And yet you want to be accepted in the program, you are still getting your social footing. You are meeting all of these new supervisors and all of these people that will be important for your life. There are like, power differential. (Trainee 16)

### 4.3 Theme 3: fostering transformation involves building confidence, agency and trust

Transformative experiences were not common but did occur: ‘I really truly learned a lot from it. And it definitely changed my view of professionalism. And it definitely makes me a little bit more cognizant now when I see professionalism lapses occur in the hospital’ (Trainee 3).

Confidence building was highlighted as essential in helping students navigate professionalism lapses, requiring both support and time to achieve. ‘I think most people … are pretty deflated and disillusioned …. So, I spend a fair bit of time trying to rebuild some of that confidence and self‐esteem’ (Faculty 5). This is particularly relevant in the clinical environment where remediation for professionalism lapses can potentiate imposter feelings:

She started to feel a bit more judged …. She viewed it as something that … was bringing her confidence down that had to be watched a little bit more closely … which then was just a cycle for the student. She just then couldn't get back any confidence at all. So, she would get quieter and meeker and wouldn't take control of the situation. And really then felt like, “I don't think that I can do this.” (Faculty 3)

Both faculty and students highlighted the value in creating student agency during professionalism processes: ‘It's important for students to have a voice in the process of addressing professionalism concerns’ (Faculty 2). Trainees noted: ‘They allowed me the chance to speak … it felt respectful … and they kind of gave me the benefit of the doubt’ (Trainee 5), and ‘there should be a stronger emphasis on really seeing from the student's point of view how they can help rather than sort of punishing somebody for making those lapses if that is the real goal’ (Trainee 7). Some processes even relied on the student's perspective ‘… all I get told is that we have a student needing remediation. And it is the student that actually shares with me what brings them to me. I don't get any other details which I actually think is really great. So, it doesn't let me have any preconceived ideas. The student can talk me through, it really helps, when you talk about dialogue, it helps that part’. (Faculty 3). Another trainee said: ‘You are expected to create your learning plan. And they are there to help you and to add things in. But it really should be driven by what you are looking to get from the process’ (Trainee 3).

Creating a safe environment where students feel comfortable sharing and reflecting builds trust and helps manage the guilt and shame involved: ‘We need to create a space where students can reflect without fear’. (Faculty 5). In such environments, students can address their lapses without feeling threatened: ‘When students feel safe, they are more open to feedback and willing to grow’. (Faculty 3). Both faculty and trainees recognised the importance of this trust when putting a ‘resident to work primarily with a small group of supervisors because he identified those supervisors as people that he trusts … that he values the feedback’ (Faculty 4) and ‘having a trusted, friendly person who you have a relationship with preferably, and who is on your side …. They can assess the validity of that accusation and hear your side and offer you support’ (Trainee 2). This trust could be transformative:

And so that was [a] big pivotal reflective moment for me, hearing those stories from other physicians …. The more you talk about it, the less shame you feel. And I think that has changed too. I think I am much more open in talking about my professionalism process and my professionalism lapse than I was before. Before I was so terrified to tell anybody. And now [I talk] about it whenever because maybe it will help somebody else. (Trainee 3)

### 4.4 Theme 4: deep perspective shift involves deep engagement over time, including but not limited to self‐reflection, structured discussion and seeking support

Perspective shift in response to professionalism lapses can be multifaceted. The remediation process can have a significant impact on a student's understanding of professionalism more broadly, and the importance of the remediation process itself: ‘And it has made me reflect a lot about professionalism in medicine. And how we think about what is considered professional …. I really have a different view of professionalism in general and how we define it’ (Trainee 3). Conversely, perspective shift can also result in superficial change to actions—being ‘scared’ into doing the ‘right’ thing to avoid getting called out again. Professionalism lapses often took time to resolve, which was perceived both as uncomfortable and necessary for deep engagement and perspective shift:

I think that in an ideal world, this would all be wrapped up in a couple of weeks because it is looming over your head. But at the same time in order to meaningfully engage with it, it took a lot of time … it is stretched over four months. And I was stressed about it for four months. But I also needed time to go through and engage and also not have it impact my program or my residency match. So, I think it was as good as it could have been. (Trainee 3)

Reflection was framed as a key part of the process, and a component that contributed to its outcome: ‘I think the educational aspect is really important. Really making people understand why those rules are the way they are. And having them reflect on them, having them reflect on their own factors is really important’ (Trainee 3). Furthermore, ‘if they can't really reflect upon that, then I am not sure if you are really going to inspire too much change’ (Trainee 14).

Discussion was highlighted as an important tool to navigate the process and encourage trainees to deeply engage in understanding the concern: ‘It was mostly just an open discussion … about what we were going to do, what has happened, and things like that. The whole process for me was just an open, one‐on‐one discussion. So I felt, like I said I felt very heard’ (Trainee 8). Discussion is important ‘especially when someone doesn't know that they actually did something wrong …. I think that when you talk to someone about something that maybe was harmful, you have the opportunity to actually have that back‐and‐forth discussion about it’ (Trainee 11).

Finally, the importance of trainee support was clear:

In general, there should always be at least one point of contact or one person that the student can talk to directly or one‐on‐one about the remediation process. And meet with them one on one …. It is a little bit of a scary process because the school presents it as like, “you have to go through this, otherwise you will [need] to repeat your year or be kicked out of the program,” right? (Trainee 8)

Accepting help and developing self‐awareness were important parts of support seeking:

And also, I think learning to be more willing to accept help because one thing from before all my meetings was, you know, I thought I don't need accommodations, I can power through this just like everyone else. And then I kind of later learned that is not the way to go about things, right? (Trainee 2)

For another trainee, ‘a big part of my learning plan was figuring out how to better manage my stress and prioritize my health so that it would prevent this professionalism lapse from happening’ (Trainee 3). The complexities of where to seek support were also highlighted:

I think having an external body to help provide an unbiased lens and just help students navigate, just to provide advice even in some of these professionalism issues would be really helpful … staff and the faculty on Trainee Affairs have multiple positions and oftentimes, they are preceptors themselves, and so, students are often hesitant to reach out to them for help and guidance. (Trainee 4)

## 5 DISCUSSION

This study uncovered complexities in the identification of and response to professionalism lapses identified by both trainees and educators. The perception and recognition of professionalism lapses was that they are subjective and shaped by faculty, institutional and situational contexts. This subjectivity can complicate the identification of and response to professionalism lapses, especially when standards are implicit or embedded within a context bound culture. Much like an umpire issuing a ‘Yellow Card’ in sport, professionalism lapses were often embedded within a power hierarchy. These power hierarchies potentially limit trainee ability to deeply engage or respond to concerns around professionalism lapses, risking defensive or superficial responses. Despite these complexities, examples of transformative learning around professionalism lapses did occur. Developing trainee confidence, agency and trust was important to set the stage for transformative experiences. Transformative experiences were linked to deep engagement with professionalism concerns through self‐reflection, structured discussion and focused support.

### 5.1 The role of theoretical frameworks

These findings support the relevance of transformative learning theory, which situates perspective change through critical reflection and discourse, as a response to disorienting dilemmas.^25^ In the context of professionalism lapses, these dilemmas hold unique potential to threaten trainee's identity, as they call into question the professional appropriateness of personal and/or social behavioural norms. Counterbalancing these threats with a process that both provides trainees support for reflection and dialogue as well as agency and voice within the process can increase the likelihood that these dilemmas catalyse personal and professional growth.

Additionally, existing power dynamics between supervisors and trainees and the inherent hierarchical structures around professionalism processes complicate trainees' navigation of a dilemma. There is potential for power dynamics to prompt defensive responses from trainees that focus on the legitimacy of the process, question the singling out of behaviours and variation inherent in professionalism standards and processes. Some of these concerns are warranted, as professionalism lapses can be ‘weaponized’ against trainees, particularly among those who are from historically underrepresented groups in medicine,^34^ to punish dissent or enforce conformity within hierarchical structures.^35^ This contributes to an environment where trainees may not feel safe to express genuine concerns or challenge existing practices, hindering reflective practices and open discourse around professionalism standards. It also has the potential to redirect focus from understanding the contextual nature of professionalism to feelings of injustice and/or inequity, limiting the transformational potential of process around professionalism lapses.

These findings are also relevant to concepts from social learning theories such as communities of practice and situated learning,^15^, ^16^, ^17^, ^18^ which emphasise learning as a social endeavour—unfolding within a community through shared practices and norms. Understanding the nuances around local group norms and expectations creates challenges for trainees rotating in and out of multiple clinical training environments with different practices. These variations in professionalism standards across different clinical contexts, cultures and institutions risk trainees feeling that standards are fluid or even idiosyncratic. Acceptable behaviours in one context, even role‐modelled by faculty, may be considered unprofessional in others.^36^ Additionally, the goal of socialising to professional expectations becomes compounded by generational and cultural differences that intersect within clinical learning environments with much left implicit and undiscussed in advance of professionalism concerns being labelled and trainees singled out.

Raising concerns around professionalism lapses with trainees also has risks, with the potential to threaten nascent professional identity development, contribute to imposter syndrome feelings and undermine confidence required for trainee engagement in clinical learning. These theories highlight the importance of integrating trainees into professional communities in ways that allow them to internalise and negotiate professional norms and identities.

### 5.2 Practical relevance for educators and trainees

For educators, these findings emphasise the need for clearly communicating professional standards—including the explicit translation of standards to clinical contexts and individualised expectations and interpretation of standards—within a clinical learning environment. When a lapse is identified, remediation processes should create space for perspectives and dialogue, recognising that varied interpretations of professionalism standards are frequent, and power dynamics can influence the process in unintended ways. Fostering trust includes the creation of a learning environment where students feel safe to discuss and learn from professionalism lapses without fear of undue punishment. This safety should leave open the possibility for a bidirectional process where discussion can influence and evolve the norms of professionalism within educational programmes. This includes (1) guarding against cultural and generational biases influencing standards and lapses and (2) a willingness to reconsider and potentially reshape understanding of professionalism to ensure that standards remain relevant and inclusive.^37^ Finally, faculty should be cognizant of the behaviour they role model and ensure deviations are discussed with contextual explanations to avoid devaluing professional standards in the eyes of trainees as part of the hidden curriculum.

For trainees, these findings highlight the transformative potential of firsthand experience with professionalism lapses, which were not only more difficult to navigate but also more impactful than hearing experiences of peers or learning about lapses in a classroom environment. Trainees should be encouraged to view professionalism lapses not as failures, but as opportunities for significant learning and growth. Clearly, there were benefits in engaging actively in structured reflection and discussions about professionalism, particularly around understanding its contextual nature. Seeking out support and feedback was important in navigating concerns, as the processes to address professionalism concerns were inherently stressful.

Practical advice for educators and trainees navigating professionalism lapses is summarised in Table 2.

**TABLE 2:** Practical advice for educators and trainees navigating professionalism lapses.

	Educators	Trainees
*Communicating professionalism standards*	Be explicit with professionalism expectations when orienting learners. Discuss standards and clarify how they apply in the clinical context for trainees. Role model standards, and use the opportunity to highlight when standards apply in practice.	Recognise that professional expectations are context specific. Ask supervisors how they view professional expectations and how they apply within their clinical context.
*Critical reflection and considerations of power around professionalism lapses*	Be clear about the standards, and explain the rationale for considering trainee behaviour a lapse. Ensure trainees are not held to a standard that faculty do not uphold and create a transparent space or process for trainees to report concerns of being held to a different standard. Create opportunities for educators to also engage in critical reflection about discussion with trusted peers about other possible interpretations of professionalism lapses. Be explicit and cognizant of the context in which the lapse occurred, and the ways in which the context contributed to the perception of the lapse.	Give time to listen and digest concern around professionalism lapse. Ask for clarity around standards and the context in which the lapse occurs. Commit to a response, but recognise that it does not have to happen in the moment.
*Promoting transformative learning around professionalism lapses*	Be transparent about the structures and processes that will be used to manage professionalism lapses including ways in which trainees can engage, support offered and potential outcomes of the process. Give trainees time, space and support to process and respond to a concern. Provide structure for reflection and discussion outside of the supervision power hierarchy. Provide opportunities for trainees to develop action plans for the future when contextual factors make it difficult to uphold professional standards.	Seek out support and alternate perspectives before responding to a professionalism lapse. Invest in critical reflection and discussion with trusted mentors and/or peers. Seek out opportunities to demonstrate understanding of professional expectations and role model professionalism standards.

### 5.3 Limitations

While this study provides valuable insights, the perspectives are self‐reported and provided in hindsight, which may understate the degree of transformation, as perspective shift may be quickly subsumed into individual ways of knowing and viewing experiences in a way that obscures previous perspective. We also recognise that our sample may not have captured unique vulnerabilities and challenges faced by historically underrepresented groups in navigating professionalism concerns; future work should seek to understand how the experience of professionalism remediation processes may vary among trainees from underrepresented populations in health care. Furthermore, this study was situated in a single institution, itself contextually bound. While sampling across programmes with diverse learning sites adds to the robustness of the findings, commonalities in culture or approach across the whole institution would not be surfaced. Of relevance, the Faculty developed a professionalism rubric^38^ relevant to all health professions, which defines four domains of professionalism (professional responsibility & integrity, pursuit of excellence and insight, personal interactions, and equity, diversity, inclusivity and indigenous reconciliation) with multiple subdomains within each. The rubric codifies behaviours inconsistent with, consistent with and exemplary of professional practice. As a result, many programmes would have a common language and shared understanding of professionalism standards, which might enable identification of lapses and facilitate conversation. Finally, some trainees were interviewed while still in their programmes; this near term reflection provides the vividness of recency but misses longer term impact as trainees migrate into professional practice.

## 6 CONCLUSION

Identifying and addressing professionalism lapses requires nuanced and contextual exploration of personal, institutional and situational dynamics at play. By fostering environments that promote genuine reflection and dialogue, health professions training programmes can better support trainees in navigating these complex situations and contribute to the broader goal of socialising to a professional culture and practice.

## AUTHOR CONTRIBUTIONS

**Matt Sibbald:** Conceptualization; funding acquisition; methodology; formal analysis; writing – original draft; supervision. **Urmi Sheth:** Writing – review and editing; data curation; investigation; formal analysis; software; methodology. **Nicole Last:** Investigation; data curation; formal analysis; writing – review and editing; software; methodology. **Amy Keuhl:** Project administration; formal analysis; writing – review and editing. **Isla McPherson:** Conceptualization; writing – review and editing. **Sarah Wojkowski:** Conceptualization; writing – review and editing. **Dorothy Bakker:** Conceptualization; writing – review and editing. **Paula Rowland:** Conceptualization; writing – review and editing.

## CONFLICT OF INTEREST STATEMENT

The authors report no conflicts of interests to declare.

## ETHIC STATEMENT

This study was approved by the Hamilton Integrated Research Ethics Board, Protocol #15055.

## Supporting information


**Appendix S1.** Supporting information.

## Data Availability

The data that support the findings of this study are available on request from the corresponding author. The data are not publicly available due to privacy or ethical restrictions.

## References

[1] The Association of Faculties of Medicine of Canada . Canadian undergraduate deans statement on professionalism. 2021. Accessed May 1, 2024. https://www.afmc.ca/wp-content/uploads/2022/10/ProfessionalismPolicyUG_Final_EN.pdf

[2] Lucey C , Souba W . Perspective: the problem with the problem of professionalism. Acad Med. 2010;85(6):1018‐1024. doi:10.1097/acm.0b013e3181dbe51f 20505405

[3] Mak‐van der Vossen M , Teherani A , van Mook W , Croiset G , Kusurkar RA . How to identify, address and report students' unprofessional behaviour in medical school. Med Teach. 2019;42(4):372‐379. doi:10.1080/0142159x.2019.1692130 31880194

[4] Findyartini A , Sudarsono NC . Remediating lapses in professionalism among undergraduate pre‐clinical medical students in an Asian institution: a multimodal approach. BMC Med Educ. 2018;18(1):88. doi:10.1186/s12909-018-1206-2 29716581 PMC5930750

[5] Mak‐Van Der Vossen M , Van Mook W , Van Der Burgt S , et al. Descriptors for unprofessional behaviours of medical students: a systematic review and categorisation. BMC Med Educ. 2017;17(1):1‐12. doi:10.1186/s12909-017-0997-x 28915870 PMC5603020

[6] Papadakis MA , Teherani A , Banach MA , et al. Disciplinary action by medical boards and prior behavior in medical school. N Engl J Med. 2005;353(25):2673‐2682. doi:10.1056/NEJMsa052596 16371633

[7] Brennan N , Price T , Archer J , Brett J . Remediating professionalism lapses in medical students and doctors: a systematic review. Med Educ. 2020;54(3):196‐204. doi:10.1111/medu.14016 31872509

[8] Parker M , Luke H , Zhang J , Wilkinson D , Peterson R , Ozolins I . The “pyramid of professionalism”: seven years of experience with an integrated program of teaching, developing, and assessing professionalism among medical students [published correction appears in *Acad Med*. 2008;83(12):1169]. Acad Med. 2008;83(8):733‐741. doi:10.1097/ACM.0b013e31817ec5e4 18667884

[9] Rabow MW , Remen RN , Parmelee DX , Inui TS . Professional formation: extending medicine's lineage of service into the next century. Acad Med. 2010;85(2):310‐317. doi:10.1097/acm.0b013e3181c887f7 20107361

[10] Byszewski A , Hendelman W , McGuinty C , Moineau G . Wanted: role models—medical students' perceptions of professionalism. BMC Med Educ. 2012;12(115):115. doi:10.1186/1472-6920-12-115 23153359 PMC3537482

[11] Haidet P , Stein HF . The role of the student‐teacher relationship in the formation of physicians. The hidden curriculum as process. J Gen Intern Med. 2006;21(Suppl 1):S16‐S20. doi:10.1111/j.1525-1497.2006.00304.x 16405704 PMC1484835

[12] Hickson GB , Pichert JW , Webb LE , Gabbe SG . A complementary approach to promoting professionalism: identifying, measuring, and addressing unprofessional behaviors. Acad Med. 2007;82(11):1040‐1048. doi:10.1097/ACM.0b013e31815761ee 17971689

[13] Dyrbye LN , Massie FS Jr , Eacker A , et al. Relationship between burnout and professional conduct and attitudes among US medical students. JAMA. 2010;304(11):1173‐1180. doi:10.1001/jama.2010.1318 20841530

[14] Roberts LW . Understanding depression and distress among medical students. JAMA. 2010;304(11):1231‐1233. doi:10.1001/jama.2010.1347 20841539

[15] Lave J , Wenger E . Situated Learning: Legitimate Peripheral Participation. Cambridge University Press; 1991. doi:10.1017/CBO9780511815355

[16] Kolb DA . Experiential Learning: Experience as the Source of Learning and Development. 2nded. FT Press; 2014.

[17] Wenger E . Communities of Practice: Learning, Meaning, and Identity. Cambridge University Press; 1998. doi:10.1017/CBO9780511803932

[18] Wenger‐Trayner E , Fenton‐O'Creevy M , Hutchinson S , Kubiak C , Wenger‐Trayner B . Learning in Landscapes of Practice: Boundaries, Identity, and Knowledgeability in Practice‐Based Learning. 1sted. Routledge; 2014. doi:10.4324/9781315777122

[19] Durning SJ , Artino AR . Situativity theory: a perspective on how participants and the environment can interact: AMEE guide no. 52. Med Teach. 2011;33(3):188‐199. doi:10.3109/0142159X.2011.550965 21345059

[20] Wenger E . Communities of practice and social learning systems. Organ. 2000;7(2):225‐246. doi:10.1177/135050840072002

[21] Mezirow J . Transformative Dimensions of Adult Learning. Jossey‐Bass; 1991.

[22] Mezirow J (Ed). Learning to think like an adult: Core concepts of transformation theory. In: Learning as Transformation: Critical Perspectives on a Theory in Progress. Jossey‐Bass; 2000:3‐33.

[23] Mezirow J . Transformative learning as discourse. J Transform Educ. 2003;1(1):58‐63. doi:10.1177/1541344603252172

[24] Greenhill J , Poncelet AN . Transformative learning through longitudinal integrated clerkships. Med Educ. 2013;47(4):336‐339. doi:10.1111/medu.12139 23488752

[25] Vipler B , Knehans A , Rausa D , Haidet P , McCall‐Hosenfeld J . Transformative learning in graduate medical education: a scoping review. J Grad Med Educ. 2021;13(6):801‐814. doi:10.4300/JGME-D-21-00065.1 35070093 PMC8672835

[26] Sutton J , Austin Z . Qualitative research: data collection, analysis, and management. Can J Hosp Pharm. 2015;68(3):226‐231. doi:10.4212/cjhp.v68i3.1456 26157184 PMC4485510

[27] Braun V , Clarke V . Thematic Analysis: A Practical Guide. Sage; 2021.

[28] Sandelowski M . Focus on research methods‐whatever happened to qualitative description? Res Nurs Health. 2000;23(4):334‐340. doi:10.1002/1098-240x(200008)23:4<334::aid-nur9>3.0.co;2-g 10940958

[29] Sandelowski M . What's in a name? Qualitative description revisited. Res Nurs Health. 2010;33(1):77‐84. doi:10.1002/nur.20362 20014004

[30] Saldana J . The Coding Manual for Qualitative Researchers. 2nded. Sage; 2013.

[31] Malterud K , Siersma VD , Guassora AD . Sample size in qualitative interview studies: guided by information power. Qual Health Res. 2016;26(13):1753‐1760. doi:10.1177/1049732315617444 26613970

[32] Kitto SC , Chesters J , Grbich C . Quality in qualitative research. Med J Aust. 2008;188(4):243‐246. doi:10.5694/j.1326-5377.2008.tb01595.x 18279135

[33] Steier F (Ed). Reflexivity and methodology: an ecological constructionism. In: Research and Reflexivity. Sage; 1991:163‐185.

[34] Maristany D , Hauer KE , Leep Hunderfund AN , et al. The problem and power of professionalism: a critical analysis of medical students' and residents' perspectives and experiences of professionalism. Acad Med. 2023;98(11S):S32‐S41. doi:10.1097/ACM.0000000000005367 37983394

[35] Borrero M , Kiel L , Abuali I , Ivy ZK , Florez N . The weaponization of professionalism against physicians of color. Hum Resour Health. 2024;22(1):52. doi:10.1186/s12960-024-00931-y 39014457 PMC11251374

[36] Martimianakis MA , Maniate JM , Hodges BD . Sociological interpretations of professionalism. Med Educ. 2009;43(9):829‐837. doi:10.1111/j.1365-2923.2009.03408.x 19674298

[37] Aguilar AE , Stupans L , Scutter S . Assessing students' professionalism: considering professionalism's diverging definitions. Educ Health (Abingdon). 2011;24(3):599. doi:10.4103/1357-6283.101417 22267355

[38] McMaster University . Professionalism in practice. 2023. Accessed July 27, 2024. https://ugme.healthsci.mcmaster.ca/wp-content/uploads/2023/05/Professionalism-in-Practice-March-2023.pdf

