# Supplementary material for: Professionalism lapses in health professions training: Navigating the ‘Yellow Card’ moments for transformative learning
Source: Med Educ. 2024 Oct 2;59(4):418–27. doi: 10.1111/medu.15540 (PMC11906273; doi:10.1111/medu.15540)
Supplement: Supplementary file 1 — Appendix S1. Supporting information. [file MEDU-59-418-s001.docx]

**Appendix S1**

**Interview Guide for Faculty/Staff**

*This interview guide is for faculty/staff who have been involved in remediating professionalism lapses among trainees in healthcare training programs at McMaster University.*

**Please Note:** This is an interview guide, not an interview script. It is anticipated that this guide will evolve as the project progresses. While the interview questions will not always remain exactly as written, nor in the same order, the interviews will proceed along the same general format. This interview guide includes questions that relate to how professionalism lapses are defined, uncovered, managed and remediated, and around components relevant to social and transformative theoretical lenses (e.g. to what extent does critical reflection occur in the process).

**THE INTERVIEW PROCESS BEGINS HERE**

**Introductions and Informed Consent** *(not recorded)*

Thank you for agreeing to participate in this interview today. We appreciate you taking the time.

*-Interviewers introduce themselves, then screenshare the Info/Consent Form.*

This is the Information and Consent Form for this study. I believe you have already received it and signed a copy, is this correct?

*[If they say no, email them the Info/Consent Form and ask them to read it, sign it electronically, and email it back to you.]*

Let’s take a moment to review some of our privacy protocols:

-We will be recording and transcribing this interview.

-The research coordinator will remove any identifying information in the transcript before the analytic team has access to it.

-In a few moments, we will ask you for some personal data. We are assigning ID numbers to each study participant, so your name will not be attached to your personal data.

-All of the personal data that we collect will be aggregated for the manuscript.

-No one on the analytic team and none of your colleagues will know you have participated in this study unless you tell them.

-We may use direct quotes from the interview transcripts in the manuscript for this study. The research team will make sure that the quotes contain no personal identifiers which would reveal your identity.

Do you have any questions about our privacy protocols or anything else in the Information and Consent form?

*[If yes, discuss. If no, continue.]*

**Psychological Safety/Pausing/Withdrawing** (not recorded)

We anticipate this interview will take approximately 60 minutes. We may take some notes while you’re talking. Please answer each question as fully as possible. There are no right or wrong ways to respond. If you would prefer not to answer a question, just say “pass”. We know that discussing professionalism lapses and remediation can be challenging. If you ever need to take a break or would like to stop the interview, please let us know. If you would like to withdraw from this study after the interview, just email me or email the research coordinator.

Do you have any questions?

**Personal Data Collection** *(not recorded)*

*[Enter this data into the Data Collection Sheet]*

We will now gather some personal information from you:

-Are you a faculty member or a staff member?

-What is your gender?

-What is your healthcare profession? (e.g. medicine, nursing, midwifery, etc.)

-What has been your involvement with student professionalism lapses and remediation processes? (multiple choice: once or twice, 3-9 times, 10 or more times)

We’re going to start recording now. Once the recording begins, please try not to say the names of any students or colleagues. If you do by accident, those names will be redacted.

**Start recording.**

**Defining a Professionalism Lapse**

What is a professionalism lapse? How would you define one?

In your opinion, what are some factors that can lead to a professionalism lapse among trainees?

Have you ever experienced a situation when you believed a trainee had a professionalism lapse, but others did not agree? Or vice versa, where another person believed a trainee had a professionalism lapse, but you did not agree? If so, please explain the situation and how it was resolved.

**Uncovering Professionalism Lapses**

Based on your experience, how are professionalism lapses uncovered? What is the role of students? Faculty? Instructors or Preceptors? Patients and families?

**The Professionalism Remediation Process**

In your opinion, what should be the goal of remediation when there is a professionalism lapse in a trainee?

Can you think of a time when a professionalism lapse was well-managed within your program or another program? If so, please explain how the process was handled and what steps were taken.

Can you think of a time when a professionalism lapse was poorly managed within your program or another program? If so, please explain what happened and what could have been done differently.

Can you suggest any ‘best practices’ or ‘wise practices’ that programs should use when remediating professionalism lapses among trainees?

**Social and Transformative Theoretical Lenses**

Professionalism lapses can often be seen as identity threatening, or disorienting. Can you think of examples where this was the case? Any examples where this was not the case?

Reflecting on professionalism lapses is often thought to be key to growth. Can you think of examples where remediation processes used reflection? Can you think of examples where the processes may have impaired or gotten in the way of reflection?

Dialogue is another tool that can be helpful in remediating reflection. How is dialogue incorporated into professionalism training or remediation in your context? Are there circumstances that make dialogue particularly effective? What role did a mentor or advisor play?

Can you think of any examples where the remediation process has fundamentally changed a trainee? If so, what do you think contributed? If not, why not?  In your opinion what would have sparked change?

Reflection, dialogue and fundamental change within a trainee all take time. In the examples you mentioned, do you have a sense of the time frame involved from professionalism concern being raised to resolution?

**Other Potential Prompt Questions** *(to be used throughout the interview)*

- Can you tell me more about that?
- Please explain further.
- What happened next?
- What was the outcome?
- Why do you think that is? What made you come to that conclusion?

**Conclusion**

Those are all the questions we have for you right now. Did we miss anything? Do you have any questions for us?

I will now stop recording. *[Stop recording]*

We will use this recording to create a transcript of the interview. We may contact you if there is a part of the recording we can’t understand, or if we need some clarification.

Thank you again for your time and for participating in this study!

**Interview Guide for Students/Graduates**

*This interview guide is for students/graduates who have firsthand or secondhand experience involved in remediating professionalism lapses among trainees in healthcare training programs at McMaster University.*

**Please Note:** This is an interview guide, not an interview script. It is anticipated that this guide will evolve as the project progresses. While the interview questions will not always remain exactly as written, nor in the same order, the interviews will proceed along the same general format. This interview guide includes questions that relate to how professionalism lapses are defined, uncovered, managed and remediated, and around components relevant to social and transformative theoretical lenses (e.g. to what extent does critical reflection occur in the process).

**THE INTERVIEW PROCESS BEGINS HERE**

**Introductions and Informed Consent** *(not recorded)*

Thank you so much for agreeing to participate in this interview today. We know that discussing professionalism lapses and remediation can be difficult, so we appreciate you taking the time.

*-Interviewers introduce themselves, then screenshare the Info/Consent Form.*

This is the Information and Consent Form for this study. I believe you have already received it and signed a copy, is this correct?

*[If they say no, email them the Info/Consent Form and ask them to read it, sign it electronically, and email it back to you.]*

Because this interview will contain personal and confidential information, we’d like to take a minute to review some of our privacy protocols:

-We will be recording and transcribing this interview.

-The research coordinator will remove any identifying information in the transcript before the analytic team has access to it.

-Before we start recording, we will ask you for some personal data. We are assigning ID numbers to each study participant, so your name will not be attached to your personal data.

-All of the personal data that we collect will be aggregated for the manuscript.

-No one on the analytic team and no one in your program, including faculty, staff, students, or graduates will know you have participated in this study unless you tell them.

-We may use direct quotes from the interview transcripts in the manuscript for this study. The research team will make sure that the quotes contain no personal identifiers which would reveal your identity.

Do you have any questions about our privacy protocols or anything else in the Information and Consent form?

*[If yes, discuss. If no, continue.]*

**Psychological Safety/Pausing/Withdrawing** (not recorded)

We anticipate this interview will take approximately 60 minutes. We may take some notes while you’re talking. Please answer each question to the best of your ability. There are no right or wrong ways to respond. If you would prefer not to answer a question, just say “pass”. We know that discussing professionalism lapses and remediation can be challenging. If you ever need to take a break or would like to stop the interview, please let us know. If you would like to withdraw from this study after the interview, just email me or email the research coordinator.

We also can provide you with a list of resources should you need help or support:

1. McMaster’s [Student Wellness Centre](https://wellness.mcmaster.ca/) (SWC) provides counselling support to students. You can book an appointment online.
2. [Good2Talk](https://good2talk.ca/) is a 24 hour-7 days a week confidential service for post-secondary students who wish to speak with a counsellor by phone or text. Call 1-866-925-5454 or text GOOD2TALKON to 686868

Do you have any questions?

**Personal Data Collection** *(not recorded)*

*[Enter this data into the Data Collection Sheet]*

We will now gather some personal information from you:

-Are you a current student at McMaster or are you a recent graduate?

-How would you describe your gender identity?

-How would you describe your race/cultural identity?

-What is/was your program? (e.g. medicine, nursing, midwifery, etc.)

-Do have firsthand experience with a professionalism remediation process, secondhand experience, or both? By ‘firsthand experience’, we mean you were involved in the process directly. By ‘secondhand experience’, we mean you know of someone who was involved in a professionalism remediation process.

We’re going to start recording now. Once the recording begins, please try not to say the names of any faculty, staff or other students or graduates. If you do by accident, those names will be redacted.

**Start recording.**

**Defining a Professionalism Lapse**

In your opinion, what is a professionalism lapse? How would you define one?

Can you name some factors that could lead to a professionalism lapse among trainees?

**The Incident - Secondhand Experience**

Are you aware of any incidents of professionalism lapses among your current or former classmates?

Did you observe either in person or online the incident, or learn about it afterwards?

If you are able to, please describe what led to the professionalism lapse and what happened during the incident without saying any names.

After you observed the incident or heard about the incident, did you tell anyone about it? Why or why not? (If yes, who did you tell and why?)

**The Incident - Firsthand Experience**

If you feel comfortable, can you tell us about your own personal experience, particularly the events that led up to the professionalism lapse?

What about this incident makes you identify it as an example of a professionalism lapse? (Explain).

Did you identify this as a professionalism lapse in the moment or after the event? (Explain.)

Now that some time has passed, do you view the incident any differently? (Explain.)

After the incident happened, did you talk to anyone about it? Why or why not? (If yes, who did you talk to and why?)

**The Remediation Process**

Now we will discuss the remediation process which took place after the professionalism lapse.

In your opinion, what should be the goal of remediation when there is a professionalism lapse in a trainee?

-For those with firsthand knowledge: Please describe what took place during the remediation process.

-For those with secondhand knowledge: Based on your knowledge, what happened during the remediation process?

Do you feel the remediation process was handled well? (Why or why not?)

What can training programs do to help support students through the remediation process?

**Social and Transformative Theoretical Lenses**

Often, professionalism lapses can be seen as identity threatening, or disorienting. Can you think of examples where this was the case? Any examples where this was not the case?

Reflection on professionalism lapses is often thought to be key to growth. Can you think of examples where remediation processes used reflection?

If yes, How was reflection used in the remediation process and over what period of time? Can you think of examples where the processes may have impaired or even gotten in the way of reflection?

Dialogue is another tool that can be helpful in remediating reflection. How was dialogue incorporated into professionalism training or remediation in your context? Are there circumstances that make dialogue particularly effective?

In your opinion, did the remediation process fundamentally change you or the person who went through the remediation process?

-If yes, did this fundamental change happen right away or over time? Were the changes sustained or transient? What do you think contributed to this fundamental change?

-If not, why not?  In your opinion what would have sparked change?

**Other Potential Prompt Questions** *(to be used throughout the interview)*

- Can you tell me more about that?
- Please explain further.
- What happened next?
- What was the outcome?
- Why do you think that is? What made you come to that conclusion?

**Conclusion**

Those are all the questions we have for you right now. Did we miss anything? Do you have any questions for us?

I will now stop recording. *[Stop the recording]*

We will use this recording to create a transcript of the interview. We may contact you if there is a part of the recording we can’t understand, or if we need some clarification.

Thank you again for your time and for participating in this study. We really appreciate it!
